# Antithrombotic Management and Long-Term Outcomes of Patients with Atrial Fibrillation. Insights from CRAFT Trial

**DOI:** 10.3390/jcm10081780

**Published:** 2021-04-19

**Authors:** Paweł Balsam, Piotr Lodziński, Monika Gawałko, Leszek Kraj, Andrzej Śliwczyński, Cezary Maciejewski, Bartosz Krzowski, Agata Tymińska, Krzysztof Ozierański, Marcin Grabowski, Janusz Bednarski, Grzegorz Opolski

**Affiliations:** 1 1st Department of Cardiology, Medical University of Warsaw, 02-091 Warsaw, Poland; pawel.balsam@wum.edu.pl (P.B.); piotr.lodzinski@wum.edu.pl (P.L.); cmaciejewski6@gmail.com (C.M.); bartekkrzowski@gmail.com (B.K.); tyminska.agata@gmail.com (A.T.); krzysztof.ozieranski@gmail.com (K.O.); marcin.grabowski@wum.edu.pl (M.G.); grzegorz.opolski@wum.edu.pl (G.O.); 2 Department of Oncology, Medical University of Warsaw, 02-091 Warsaw, Poland; leszekkraj@gmail.com; 3 Satellite Campus in Warsaw, University of Humanities and Economics in Łódź, 90-212 Łódź, Poland; andrzej.sliwczynski.ahe@gmail.com; 4 Cardiology Unit, St. John Paul II Western Hospital, 05-825 Grodzisk Mazowiecki, Poland; januszbednarski4@gmail.com

**Keywords:** anticoagulation, arrhythmia, dabigatran, rivaroxaban, risk scores

## Abstract

Background: We aimed to compare long-term outcomes in Polish patients with atrial fibrillation (AF) according to oral anticoagulation (OAC) type and to evaluate the predictive value of common thromboembolic and bleeding risk scores. Methods: Data from the CRAFT trial (NCT02987062) were included. The primary study endpoint was major adverse event (MAE; all-cause death, thromboembolic and hemorrhagic event) during the mean four-year follow-up period. Results: Out of 2983 patients with available follow-up data, 1686 (56%) were prescribed with vitamin K antagonist (VKA), 891 (30%) with rivaroxaban and 406 (14%) with dabigatran. Predominance of elderly and female patients with previous history of thromboembolic and hemorrhagic events was observed within rivaroxaban (vs. other OAC) group. Higher rate of MAEs and its components was observed in patients on VKA followed by rivaroxaban as compared to patients on dabigatran (43% vs. 42% vs. 31%, *p* < 0.01). After group matching based on clinical characteristics, higher risk of hemorrhagic events in VKA (vs. dabigatran) and rivaroxaban (vs. dabigatran) group were observed. The available thromboembolic (CHA_2_DS_2_-VASs, ATRIA, R_2_CHADS_2_) and bleeding (HAS-BLED, ATRIA, ORBIT) risk scores showed poor prediction value. Conclusions: Despite no difference in the thromboembolic event rate, treatment with VKA and rivaroxaban was associated with a significant increase in the risk of hemorrhagic events.

## 1. Introduction

Oral anticoagulation (OAC) therapy can prevent the majority of ischemic strokes in patients with atrial fibrillation (AF) and can prolong life [1]. The net clinical benefit is almost universal, with the exception of patients at very low stroke risk, and OAC should therefore be used in most patients with AF [1]. Long-term OAC with a vitamin K antagonists (VKAs) or non-VKA OACs (NOACs) [2] conveys benefits in AF patients who survived a stroke with slightly better outcomes, mainly driven by fewer intracranial hemorrhages and hemorrhagic strokes [2]. Persistence to NOAC therapy is generally higher than to VKA, being facilitated by a better pharmacokinetic profile of NOACs. A recent meta-analysis showed similar reduction in ischemic stroke within patients treated with NOACs as compared to those on VKAs. Although, there was observed a significant reduction in intracranial hemorrhage among patients treated with NOACs as compared to those on VKAs, simultaneous increase in gastrointestinal bleeding was observed among patients on NOAC (vs. VKA) treatment [3]. Recent studies underlined also heterogeneity of NOAC group according to their impact on ischemic and hemorrhagic events. Significantly higher risk of major bleeding is reported for rivaroxaban than with dabigatran therapy, as was all-cause mortality and gastrointestinal bleeding with similar rate of stroke or systemic embolic events in those subgroups [4].

Therefore, the aim of the study was to compare long-term outcomes in Polish patients with AF according to OAC type (VKA vs. rivaroxaban vs. dabigatran). Moreover, we evaluated the predictive value of common thromboembolic and bleeding risk scores.

## 2. Materials and Methods

### 2.1. Study Population

This retrospective observational cohort study included data from the MultiCenter expeRience in AFib patients Treated with oral anticoagulation (CRAFT; NCT02987062). Details about the study design and main results have been reported elsewhere [5]. Briefly, CRAFT included patients aged ≥18 years, with AF, hospitalized between 2011–2016 at two academic and district hospitals. Due to retrospective character of the study, the approval of a local ethics committee and patient provided written informed consent were waived.

### 2.2. Primary and Secondary Endpoints

Primary endpoint was assigned as major adverse events (MAEs) defined as all-cause death, thromboembolic events and hemorrhagic events during follow up period of mean four years. The secondary endpoint was defined as component of primary endpoint. Data of long-term outcomes were obtained from Polish National Health Fund that gathers data about medical services that it finances, e.g., exact dates of provision, voivodeship, setting (emergency department, hospital, family medicine, outpatient clinic), primary diagnosis (International Classification of Disease—10 codes; each medical service has 1 primary diagnosis assigned), procedures (International Classification of Disease—9 codes). Ischemic events consist of diagnosis codes for: ischemic stroke (different locations), transient ischemic attack and peripheral thromboembolism (different locations). Bleeding events include gastrointestinal, intracranial and other locations of bleeding related codes. Table S1 presents list of the codes applied in the study.

### 2.3. Assessment of Bleeding Risk Scores

The HAS-BLED score was calculated by adding 1 point for each of the following factors: hypertension (uncontrolled blood pressure, systolic blood pressure > 160 mm Hg), abnormal renal function (dialysis, transplant, creatinine > 2.26 mg/dL), abnormal liver function (cirrhosis, bilirubin > 2× normal, aspartate/alanine transaminase/alkaline phosphatase > 3× normal), previous stroke, history and/or predisposition to bleeding, labile international normalized ratio (time in therapeutic range < 55%; depending on local practices, fixed doses of VKA), age > 65 years, concomitant drugs (antiplatelet agents, nonsteroidal anti-inflammatory drugs), alcohol overconsumption (≥8 drinks/week). A HAS-BLED score of 0–2 was categorized as “low risk”, and a HAS-BLED ≥ 3 was categorized as “high risk”.

The ATRIA bleeding score was calculated using the following risk factors: anemia (hemoglobin < 13 g/dL in men and <12 g/dL in women) (3 points), severe renal disease (estimated glomerular filtration rate (eGFR) < 30 mL/min/1.73 m^2^) (3 points), age ≥ 75 years (2 points), prior bleeding, and hypertension. An ATRIA score of 0–3 is defined as “low risk”, a score of 4 is defined as “intermediate risk”, and a score ≥ 5 is defined as “high risk”.

The ORBIT score was calculated as follows: age ≥ 75 years (1 point), renal dysfunction (eGFR < 60 mL/min/1.73 m^2^) (1 point), treatment with any antiplatelet (1 point), clinical history for bleeding and the presence of anemia (2 point). An ORBIT score of 0–2 was “low risk”, a score of 3 was “intermediate risk”, and a score ≥ 4 was “high risk”.

Punctation for individual score is presented in Table 1.

### 2.4. Assessment of Thromboembolic Risk Scores

The CHA_2_DS-VA_2_Sc score was calculated by adding 1 point for each of the following factors: congestive heart failure, hypertension, age 65–74, diabetes mellitus, vascular disease, female sex; and 2 points for age ≥ 75 and ischemic stroke/transient ischemic attack.

The R_2_CHADS_2_ score was calculated by adding 2 points for renal dysfunction (eGFR < 60 mL/min/1.73 m^2^) and ischemic stroke and/or transient ischemic attack (TIA); and 1 point for each of the following factors: congestive heart failure, hypertension, age ≥ 75 and diabetes mellitus.

For CHA_2_DS-VA_2_Sc and R_2_CHADS_2_, scores patients with 0 point were defined as being in the low-risk category and patients with 1 point were at intermediated risk, while patients with ≥2 points were in the high-risk stratum.

The ATRIA thromboembolic risk score was calculated by adding 1 point for each of the following factors: female sex, diabetes mellitus, congestive heart failure, hypertension and renal dysfunction (eGFR < 45 mL/min/1.73 m^2^) and by adding 0–9 points depending on the specific score weighting of patients age according to the presence or absence of prior ischemic stroke. We did not have data about proteinuria, so the maximum score of the ATRIA thromboembolic risk score will be 14 points. Patients with ≤5 points were defined as low-risk category and patients with 6 points were at intermediated risk, while patients with ≥7 points were in the high-risk stratum.

Punctation for individual score is presented in Table 1.

### 2.5. Statistical Analysis

All continuous variables were tested for normality with the Shapiro–Wilk test. Variables with normal distribution were expressed as mean ± standard deviation. Nonparametric variables were expressed as median interquartile range and categorical variables as counts with percentages. Fisher’s exact test (two group comparison) or Chi-square test (three or more group comparison) were used to compare categorical variables. Differences in continuous parameters were compared using Mann–Whitney *U* test (two group comparison) and Kruskal–Wallis test (three groups comparison).

To adjust for potential confounding due to baseline imbalances in study covariates while preserving sample size, we used propensity score matching [6]. With this method, the propensity score (dabigatran or rivaroxaban treatment given baseline characteristics) was used to generate patient-specific stabilized weights that control for covariate imbalances. Covariate balance between the weighted cohorts was assessed using standardized mean differences. A standardized difference of 0.05 or less indicates a negligible difference between groups. The distributions of propensity scores and stabilized weights were inspected for outliers. All analyses (expect Table 2) were based on propensity score matching-adjusted cohorts and therefore accounted for potential confounding by baseline factors. Weighted Cox proportional hazards regression with robust estimation was used to estimate time-to-primary endpoint event in rivaroxaban compared with dabigatran (reference), VKA compared with dabigatran (reference) and VKA compared with rivaroxaban (reference) cohorts. All significance was determined using 95% confidence intervals and 2-tailed *p* values (*p* < 0.05). Weighted Kaplan–Meier analysis was used to establish the relation of type of OAC (dabigatran vs. rivaroxaban, VKA vs. rivaroxaban, VKA vs. dabigatran) to MAE and its components, and differences in adverse events were analyzed using the log-rank test.

Subgroup-specific adjusted hazard ratios with 95% CI (confidence interval) analyses were performed for all out-comes in categories defined by age, sex, heart failure, hypertension, coronary artery disease, diabetes, chronic kidney disease, HAS-BLED score and reduced dose (in case of NOAC comparison).

Evaluation of thromboembolic and bleeding risk prediction scores for patients presenting with thromboembolic and hemorrhagic events was performed utilizing the receiver operating characteristic (ROC) curve. Area under the curve (AUC) of 0.5 suggested no discrimination (ability to diagnose patients with and without of the condition base on the test), 0.5 to 0.7 was considered poor discrimination, 0.7 to 0.8 was considered acceptable, 0.8 to 0.9 was considered excellent, and more than 0.9 was considered outstanding [7].

Statistical significance was assumed at a 5% level. For database management and statistical analysis, we used SAS 14.1 (SAS Institute Inc., Cary, NC, USA).

## 3. Results

### 3.1. Study Population

Out of entire cohort of 3528 patients included in the CRAFT study, follow-up data from Polish National Health Fund was available for 3307 individuals. Out of them 2983 patients had indications for chronic anticoagulation treatment for AF in accordance with 2016 European Society of Cardiology guidelines for AF management [1] (the rest of the cohort had transient indications for anticoagulation, e.g., before and after cardioversion/ ablation). Among them, 1686 (56%) were prescribed with VKA, 891 (30%) with rivaroxaban and 406 (14%) with dabigatran.

### 3.2. Baseline Characteristics

Before propensity score matching, there were differences in demographics, comorbidity rate, and prior use of antiplatelet drugs, and calcium channel blockers between OAC groups. Patients on rivaroxaban were older, with higher predominance of female, and ischemic and hemorrhagic events compared to VKA and dabigatran groups. VKA-treated patients had more often chronic kidney disease, hence lower levels of kidney function parameters and hemoglobin concentrations (Table 2). After propensity score matching, the cohorts were well balanced across all covariates (Tables S2–S4).

### 3.3. Follow Up Outcomes Regarding OAC Type

During follow-up, there were 1128 (41%) primary outcome events including 828 (28%) deaths, 273 (9.2%) thromboembolic events and 445 (15%) hemorrhagic events. Before propensity score matching, patients treated with rivaroxaban experienced, similar to VKA group but more than dabigatran group, MAE (42% vs. 43% vs. 31%; *p* < 0.01) including all-cause death (28% vs. 29% vs. 22%; *p* < 0.01). The higher rate of hemorrhagic events was observed in VKA as compared to rivaroxaban and dabigatran groups (17% vs. 14% and 8.1%, *p* < 0.01). No statistically significant difference according to thromboembolic events were observed within rivaroxaban, dabigatran and VKA groups (10% vs. 7.4% vs. 9.0%, *p* = 0.22) (Table 2).

**Figure 1:**
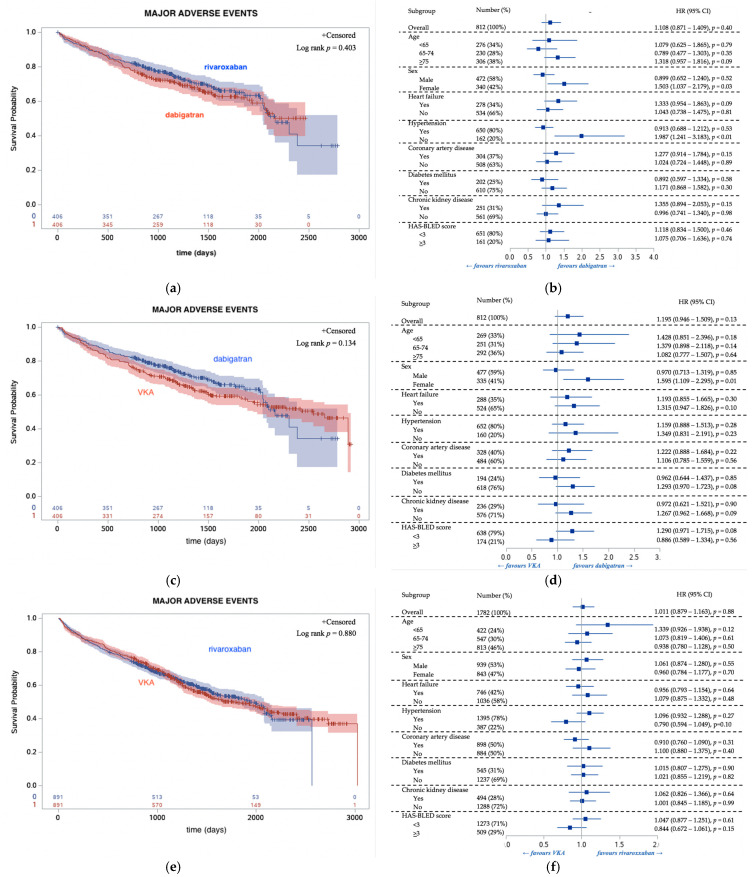
Kaplan–Meier analysis of time-to- major adverse events (left side, (**a**,**c**,**e**)) and subgroup-specific hazard ratios with 95% confidence intervals for major adverse events (right side, (**b**,**d**,**f**)) in patients treated with dabigatran vs. rivaroxaban (upper panel, (**a**,**b**)) dabigatran vs. VKA (middle panel, (**c**,**d**)) rivaroxaban vs. VKA (lower panel, (**e**,**f**)) after propensity score matching. Abbreviations: CAD, coronary artery disease; CI, confidence interval; CKD, chronic kidney disease; DM, diabetes mellitus; HF, heart failure; HR, hazard ratio; HT, hypertension; VKA, vitamin K antagonist.

After propensity score matching, statistically nonsignificant difference in MAE and its components was observed between OAC groups (Figure 1), expect statistically significant increase in hemorrhagic events in case of VKA (vs. dabigatran) and rivaroxaban (vs. dabigatran) (Figure S1).

Hazard ratios were generally consistent among subgroups (Figure 1) with a few exceptions. For thromboembolic and hemorrhagic events, the increased risk with rivaroxaban treatment (vs. dabigatran) was observed in patients aged 75 or more. Coronary artery disease, no hypertension and no diabetes status as well use of standard doses favored dabigatran (vs. rivaroxaban) against hemorrhagic events. The increased risk of thromboembolic events was observed in VKA (vs. dabigatran) in female and elderly patients, whereas age of less than 75 favored dabigatran (vs. VKA) against hemorrhagic events. Significant differences were observed within subgroups while comparing VKA and dabigatran (Figure S1).

### 3.4. Reduced and Standard Doses of NOACs

Reduced (vs. standard) doses of NOAC were consistently associated with higher rate of MAE including all-cause death and hemorrhagic events (Table 3). Moreover, reduced (vs. standard) dose of rivaroxaban was associated with higher risk of thromboembolic events. Noteworthy, 66% of both patients treated with reduced dabigatran and reduced rivaroxaban doses had recommended indications for dose reduction, whereas 12% of patients on standard dabigatran and 7.0% of patients on standard rivaroxaban despite recommended indications for dose reduction, received full doses (Table S5). Rivaroxaban-treated patients with incorrect dose prescription had higher risk of all-cause death as compared to those with correct dose prescription (Table S6).

### 3.5. Thromboembolic and Bleeding Risk Scores

**Figure 2:**
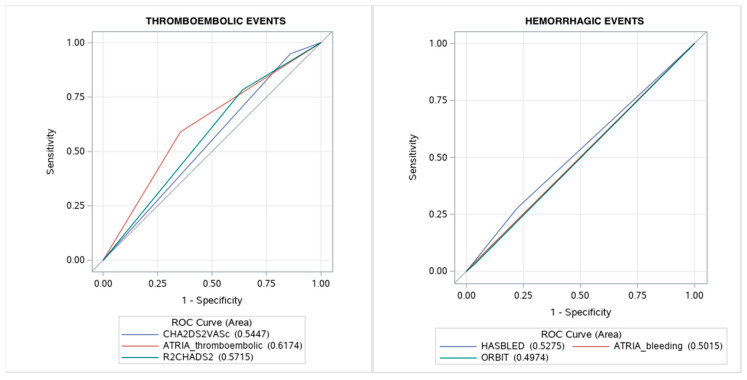
Predictive value of thromboembolic and bleeding risk scores. Abbreviations: ROC, receiver operating characteristic.

All bleeding and thromboembolic risk prediction scores demonstrated poor predictive ability for corresponding outcomes, although the HAS-BLED and ATRIA thromboembolic scores performed better than the other risk scores (Figure 2). Interestingly, HASBLED score had better prediction accuracy for thromboembolic events than CHA2DS2-VASc score in AF patients (Figure S2).

## 4. Discussion

The CRAFT study provides important data on the actual clinical practice of AF treatment among patients treated in Polish academic and district hospitals. The major findings of this study are as follows. First, in weighted OAC populations, despite no difference in the thromboembolic event rate, treatment with VKA and rivaroxaban was associated with a significant increase in the risk of hemorrhagic events. Second, the reduced (vs. standard) doses of rivaroxaban were associated with higher rate of thromboembolic events and reduced (vs. standard) doses of both dabigatran and rivaroxaban were associated with higher risk of hemorrhagic evens. Finally, the available thromboembolic and bleeding risk scores performed poor prediction value.

Before propensity score matching, the rate of MAE within the rivaroxaban group, comparable to VKA and higher than the dabigatran group, could be explained by a much older group of people burdened with higher index of previous thromboembolic and hemorrhagic events within the rivaroxaban group. It is also justified by the fact that, after propensity score matching, all of the OAC groups did not vary according to long-term outcomes, except higher rate of hemorrhagic events in the VKA (vs. dabigatran) and rivaroxaban (vs. dabigatran) group. Our results are consistent with the available literature. For the prevention of ischemic stroke, the NOACs had similar efficacy to VKA, which itself is very effective in this regard and reduces ischemic stroke by two-thirds compared with placebo [8]. NOACs are also associated with a significant reduction in all cause-mortality compared with VKA [3]. However, after group adjustment based on baseline characteristics, significance lost its statistical value. Our results are in accordance with studies comparing rivaroxaban and dabigatran. In study by Graham et al., treatment with rivaroxaban was associated with statistically significant increase in intracranial hemorrhage and major extracranial bleeding with similar rate of thromboembolic events [9]. The study by Yao et al. [10] presented rivaroxaban and dabigatran equal to VKA in ischemic events prevention and decreased risk of bleeding for dabigatran and equal for rivaroxaban. ARISTOPHANES study [11], the largest observational study to date that included 400,000 patients evaluating NOACs and VKA. In this study all NOACs were associated with lower ischemic risk than VKA and lower risk of major bleeding except for rivaroxaban which was associated with higher rate of major bleeding than VKA. No significant differences between dabigatran and rivaroxaban were noted in terms of ischemic events, however, dabigatran had significantly reduced risk of bleeding in comparison to rivaroxaban. The possible explanation of this phenomena is rivaroxaban pharmacokinetics and dosing regimen. The half-life of rivaroxaban is 5–9 h in young and 11–13 h in older population and in other indications rivaroxaban regimen is twice daily. One of the explanations of higher bleeding rate is the peak plasma concentration of rivaroxaban within first hours. This data seems to be consistent with real world data published by Graham et al. [9] and Larsen et al. [12] Hernandez et. al. [13] also reported lower ischemic risk for all NOACs, higher bleeding risk for rivaroxaban than VKA as well as than dabigatran. In this propensity matched cohort in the study from Lip et al. [14], dabigatran and apixaban but not rivaroxaban reduced risk of severe bleeding comparing to VKA. However, there was no significant difference regarding bleeding risk between dabigatran and rivaroxaban. Other studies suggested that stroke risk might be even higher with rivaroxaban use but with no difference in bleeding risks between those two NOACs [15]. This highlights the importance of head-to-head studies when evaluating comparative effectiveness of competing therapies. Our results need to be confirmed by findings from NOAC randomized control trials which are expected to be released in the upcoming years including Comparison of Efficacy and Safety Among Dabigatran, Rivaroxaban, and Apixaban in Non-Valvular Atrial Fibrillation (NCT02666157) and The Danish Non-Vitamin K Antagonist Oral Anticoagulation Study: A Cluster Randomized Study Comparing Safety and Efficacy of Edoxaban, Apixaban, Rivaroxaban and Dabigatran for Oral Anticoagulation in Atrial Fibrillation (DANNOAC-AF; NCT03129490).

The reduced (vs. standard) doses of rivaroxaban were associated with higher rate of thromboembolic events and reduced (vs. standard) doses of both dabigatran and rivaroxaban were associated with higher risk of hemorrhagic events. It is in line with the recent meta-analysis by Wang et al., that reported an elevated risk of thromboembolic and hemorrhagic events within patients eligible for reduced dose NOACs. NOACs, when appropriately dose-adjusted, had an improved benefit-harm profile compared with VKA [16]. Potential explanation of this mechanism is the fact that patients prescribed reduced dose of NOAC have lower creatinine clearance and renal function in this group is less stable. Therefore, even short periods of dehydration may result in glomerular filtration deterioration thus adverse response to OAC [17].

Various bleeding and thromboembolic risk prediction schemes have been utilized in patients with AF to assess bleeding risk. So far there is no consensus as to which score is the most appropriate. Our results according to poor prediction value of thromboembolic (CHA2DS2-VASc, ATRIA (thromboembolic), R2CHADS2) and bleeding (HASBLED, ATRIA (haemorrhagic), ORBIT) are in line with available data [18,19]. Although guidelines recommend using CHA_2_DS_2_-VASc scale in stroke risk assessment it is a kind of compromise between simplicity and practicality against precision. More complex clinical scores (ATRIA) improve stroke risk prediction especially in patients initially classified as a low risk and those with a single non-sex CHA_2_DS_2_-VASc risk factor. In a study by Abumuaileq et al., the CHA2DS2-VASc score had a better association with thromboembolic events than R2CHADS2 and ATRIA (thromboembolic) risk scores in the non-anticoagulated cohort [19], which could explain its low predictive value in our cohort of anticoagulated patients. Interestingly, even the HAS-BLED score had better prediction accuracy for thromboembolic events than the CHA2DS2-VASc score in AF patients, which is in accordance with a previous study by Roldan et al. [20]. This might reflect the need for future studies with large cohorts of patients for further validation of the available risk scores to improve actual prediction model in anticoagulated AF patients.

### Limitations of the Study

This retrospective study has several limitations. Firstly, it was not a nationwide registry with a truly representative cohort of AF patients and only inpatients were included in the registry. Secondly, long-term outcomes were based on ICD (implantable cardioverter defibrillator) codes without further adjudication using precise clinical criteria or further validation against healthcare providers’ medical records. Some publications suggest that utilization of billing codes in assessment of clinical events may overestimate their incidence rate [21]. We tried to minimize such risk in two ways. We limited the analysis only to hospital and emergency department affairs. Furthermore, we compared outcomes within groups by means of the same methodology therefore even if some overestimation cannot be ruled out, this does not affect the validity of conclusion about difference of outcomes between groups analyzed. Thirdly, our registry is limited by the fact that it depends on the data obtained from cardiology departments only. Fourthly, we are limited by information regarding anticoagulation changes during long term follow-up. Finally, due to the small number of patients on apixaban and no availability of edoxaban in Poland, they were not included into the analysis.

## 5. Conclusions

Despite no difference in the thromboembolic event rate between OACs, treatment with VKA and rivaroxaban was associated with a significant increase in the risk of hemorrhagic events in propensity matched cohorts. Our findings highlight the importance of prescribing reduced dose NOACs for indicated patient populations.

## Figures and Tables

**Table 1:** Thromboembolic and bleeding risk scores. ^a^ All patients were given a value of 0 for the proteinuria risk factor in the present study. Abbreviations: eGFR, estimated glomerular filtration rate; INR, international normalized ratio; NSAIDs, nonsteroidal anti-inflammatory drugs; TIA, transient ischemic attack.

	Thromboembolic Risk Scores			Bleeding Risk Scores		
Variable	CHA2DS2-VASc	ATRIA (Thromboembolic Risk)	R2CHADS2	HAS-BLED	ATRIA (Bleeding Risk)	ORBIT
Heart failure	1	1	1			
Hypertension	1	1	1	1	1	
Age > 85 years	2	6 or 9 (if stroke)	1	1	2	1
Age 75–84 years		5 or 7 (if stroke)				
Age 65–74 years	1	3 or 7 (if stroke)				
Diabetes mellitus	1	1	1			
Ischemic stroke/TIA	2	8	2	1		
Vascular disease	1					
Female sex	1	1				
eGFR < 60 mL/min/1.73 m^2^			2			1
eGFR < 45 mL/min/1.73 m^2^		1				
eGFR < 30 mL/min/1.73 m^2^				1	3	
Liver impairment				1		
Labile INR				1		
Excess alcohol usage				1		
Drugs (antiplatelet drugs, NSAIDs)				1		
Antiplatelet drugs						1
Prior bleeding				1	1	2
Low hemoglobin					3	2
**Results**						
Low risk	0	0–5	0	0–1	0–3	0–2
Intermediate risk	1	6	1	2	4	3
High risk	2–9	7–14 ^a^	2–8	3–9	5–10	4–7

**Table 2:** Baseline characteristics.

Variable	Rivaroxaban (*n* = 891)	Dabigatran (*n* = 406)	VKA (*n* = 1686)	*p*-Value
**Demographics**				
Age, years, median (IQR)	74 (65–81)	69 (62–78)	68 (61–78)	<0.01
Females, *n* (%)	420 (47%)	166 (41%)	638 (38%)	<0.01
**Atrial Fibrillation Type, *n* (%)**				
Paroxysmal	467 (56%) *n = 831*	208 (53%) *n = 391*	822 (51%) *n = 1622*	0.02
Long-standing persistent	8 (1.0%) *n = 831*	10 (2.6%) *n = 391*	81 (5.0%) *n = 1622*	<0.01
Persistent	99 (12%) *n = 831*	78 (20%) *n = 391*	203 (13%) *n = 1622*	<0.01
Permanent	257 (31%) *n = 831*	95 (24%) *n = 391*	516 (32%) *n = 1622*	<0.01
**Comorbidities, *n* (%)**				
Heart failure	381 (43%)	149 (37%) *n = 404*	655 (39%) *n = 1683*	0.07
Hypertension	689 (77%) *n = 890*	326 (80%)	1348 (80%) *n = 1683*	0.24
Coronary artery disease	447 (15%)	150 (39%)	759 (45%)	<0.01
Diabetes mellitus	274 (31%) *n = 887*	94 (23%) *n = 405*	486 (29%) *n = 1678*	0.02
History of TEs	157 (18%) *n = 889*	58 (14%) *n = 404*	208 (12%) *n = 1682*	<0.01
History of HEs	98 (11%)	30 (7.4%)	113 (6.7%)	<0.01
COPD	114 (13%) *n = 890*	28 (6.9%) *n = 405*	142 (8.4%) *n = 1683*	<0.01
CKD	123 (5.4%) *n = 576*	49 (17%) *n = 285*	329 (23%) *n = 1409*	0.07
Smoking	57 (6.4%)	32 (7.9%) *n = 405*	76 (4.5%) *n = 1677*	0.01
Device therapy (PM, ICD, CRT)	237 (27%)	84 (21%)	456 (27%)	0.03
**Laboratory Parameters**				
Hemoglobin, g/dL, median (IQR)	14 (13–15) *n = 574*	14 (13–15) *n = 285*	14 (13–15) *n = 1399*	<0.01
Platelet count (thousand/mm^3^, median (IQR))	205 (172–242) *n = 574*	210 (174–248) *n = 285*	202 (166–237) *n = 1403*	0.01
eGFR ≤ 14 (mL/min/1.73 m^2^), *n* (%)	3 (0.4%) *n = 831*	0 (0%) *n = 353*	3 (0.3%); *n = 1174*	<0.01
eGFR 15–29 (mL/min/1.73 m^2^), *n* (%)	18 (2.2%) *n = 831*	3 (0.9%) *n = 353*	56 (4.8%) *n = 1174*	<0.01
eGFR 30–49 (mL/min/1.73 m^2^), *n* (%)	178 (21%) *n = 831*	67 (19%) *n = 353*	312 (27%) *n = 1174*	<0.01
eGFR ≥ 50 (mL/min/1.73 m^2^), *n* (%)	632 (76%) *n = 831*	283 (80%) *n = 353*	803 (68%) *n = 1174*	<0.01
**Thromboembolic and Bleeding Scores**				
CHA_2_DS_2_-VASc score, median (IQR)	4 (3–5)	3 (2–5)	3 (2–5)	<0.01
HAS-BLED score, median (IQR)	2 (1–3)	2 (1–2)	2 (1–2)	0.06
**Other medications** **, *n* (%)**				
Antiplatelet drugs	94 (11%)	34 (8.4%)	307 (18%)	<0.01
Beta-blockers	478 (83%) *n = 576*	230 (81%) *n = 285*	1199 (85%) *n = 1409*	0.13
Calcium channel blockers	162 (28%) *n = 576*	68 (24%) *n = 285*	294 (21%) *n = 1409*	<0.01
Antiarrhythmic drugs	163 (18%)	74 (18%) *n = 405*	274 (16%) *n = 1684*	0.35
RAS inhibitors	457 (79%) *n = 576*	225 (79%) *n = 285*	1186 (83%) *n = 1410*	0.12
Statins	397 (69%) *n = 576*	172 (60%) *n = 285*	970 (69%) *n = 1410*	0.02
**Long Term Outcomes, *n* (%)**				
MAEs	373 (42%)	126 (31%)	729 (43%)	<0.01
All-cause death	250 (28%)	89 (22%)	489 (29%)	0.02
TEs	92 (10%)	30 (7.4%)	151 (9.0%)	0.22
HEs	128 (14%)	33 (8.1%)	284 (17%)	<0.01

Number provided in italic indicates the total number of patients available for that variable. Abbreviations: CKD, chronic kidney disease; COPD, chronic obstructive pulmonary disease; CRT, cardiac resynchronization therapy; eGFR, estimated glomerular filtration rate; HEs, hemorrhagic events; ICD, implantable cardioverter defibrillator; IQR, interquartile range; MAE, major adverse events; PM, pacemaker; RAS, renin–angiotensin system; TEs, thromboembolic events; VKA, vitamin K antagonist.

**Table 3:** Analysis of time-to- thromboembolic and hemorrhagic events for patients hospitalized in rivaroxaban standard and reduced doses (**A**) dabigatran standard and reduced doses (**B**) dabigatran standard and rivaroxaban standard doses (**C**) dabigatran reduced and rivaroxaban reduced doses (**D**) treatment group after propensity score matching (n = 406).

	Major Adverse Event	All-Cause Death	Thromboembolic Events	Hemorrhagic Events
	HR (95% CI)	HR (95% CI)	HR (95% CI)	HR (95% CI)
**(A) Dabigatran Standard and Reduced Doses**				
Rivaroxaban reduced (*n* = 131)	2.242 (1.608–3.125)	3.044 (1.973–4.697)	2.340 (1.180–4.637)	1.757 (1.020–3.026)
Rivaroxaban standard (*n* = 275)	reference	reference	reference	reference
**(B) Rivaroxaban Standard and Reduced Doses**				
Dabigatran reduced (*n* = 177)	2.793 (1.935–4.032)	4.716 (2.887–7.703)	0.737 (0.345–1.576)	2.034 (1.019–4.060)
Dabigatran standard (*n* = 229)	reference	reference	reference	reference
**(C) Dabigatran Standard and Rivaroxaban Standard Doses**				
Rivaroxaban standard (*n* = 275)	1.428 (0.985–2.071)	1.377 (0.807–2.350)	0.680 (0.356–1.298)	1.922 (1.026–3.602)
Dabigatran standard (*n* = 229)	reference	reference	reference	reference
**(D) Dabigatran Reduced and Rivaroxaban Reduced Doses**				
Rivaroxaban reduced (*n* = 131)	1.131 (0.817–1.567)	0.898 (0.616–1.310)	2.149 (0.975–4.737)	1.606 (0.869–2.969)
Dabigatran reduced (*n* = 177)	reference	reference	reference	reference

Abbreviations: CI, confidence interval; HR, hazard ratio.

## Data Availability

Not applicable.

## Supplementary Materials

The following are available online at https://www.mdpi.com/article/10.3390/jcm10081780/s1, Figure S1. Kaplan-Meier analysis of time-to-events (left side) and subgroup-specific hazard ratios with 95% confidence intervals for events (right side) in patients treated with dabigatran vs rivaroxaban (upper panel) dabigatran vs. VKA (middle panel) rivaroxaban vs. VKA (lower panel) after propensity score matching, Figure S2. Thromboembolic and bleeding risk scores and their predictive value. Table S1. Analyzed ICD-10 codes, Table S2. Comparison of rivaroxaban and dabigatran treatment groups after propensity score matching, Table S3. Comparison of vitamin K antagonist and dabigatran treatment groups after propensity score matching, Table S4. Comparison of rivaroxaban and vitamin K antagonist treatment groups after propensity score matching, Table S5. Distribution of direct oral anticoagulants according the recommendation for patients receiving reduced (A) and standard (B) dosing, Table S6. Analysis of time-to-major adverse events, all-cause death, thromboembolic and hemorrhagic events for patients treated with appropriate and inappropriate reduced and/or standard NOAC doses.
