# Supplementary material for: Antithrombotic Management and Long-Term Outcomes of Patients with Atrial Fibrillation. Insights from CRAFT Trial
Source: J Clin Med. 2021 Apr 19;10(8):1780. doi: 10.3390/jcm10081780 (PMC8073861; doi:10.3390/jcm10081780)
Supplement: Supplementary file 1 [file jcm-10-01780-s001.zip › jcm-1154722-supplementary.pdf]

Supplementary material online

Figure S1. Kaplan-Meier analysis of time-to-events (left side) and subgroup-specific hazard ratios with 95% confidence intervals for events (right side) in patients treated with dabigatran vs rivaroxaban (upper panel) dabigatran vs VKA (middle panel) rivaroxaban vs VKA (lower panel) after propensity score matching.

A) All-cause death

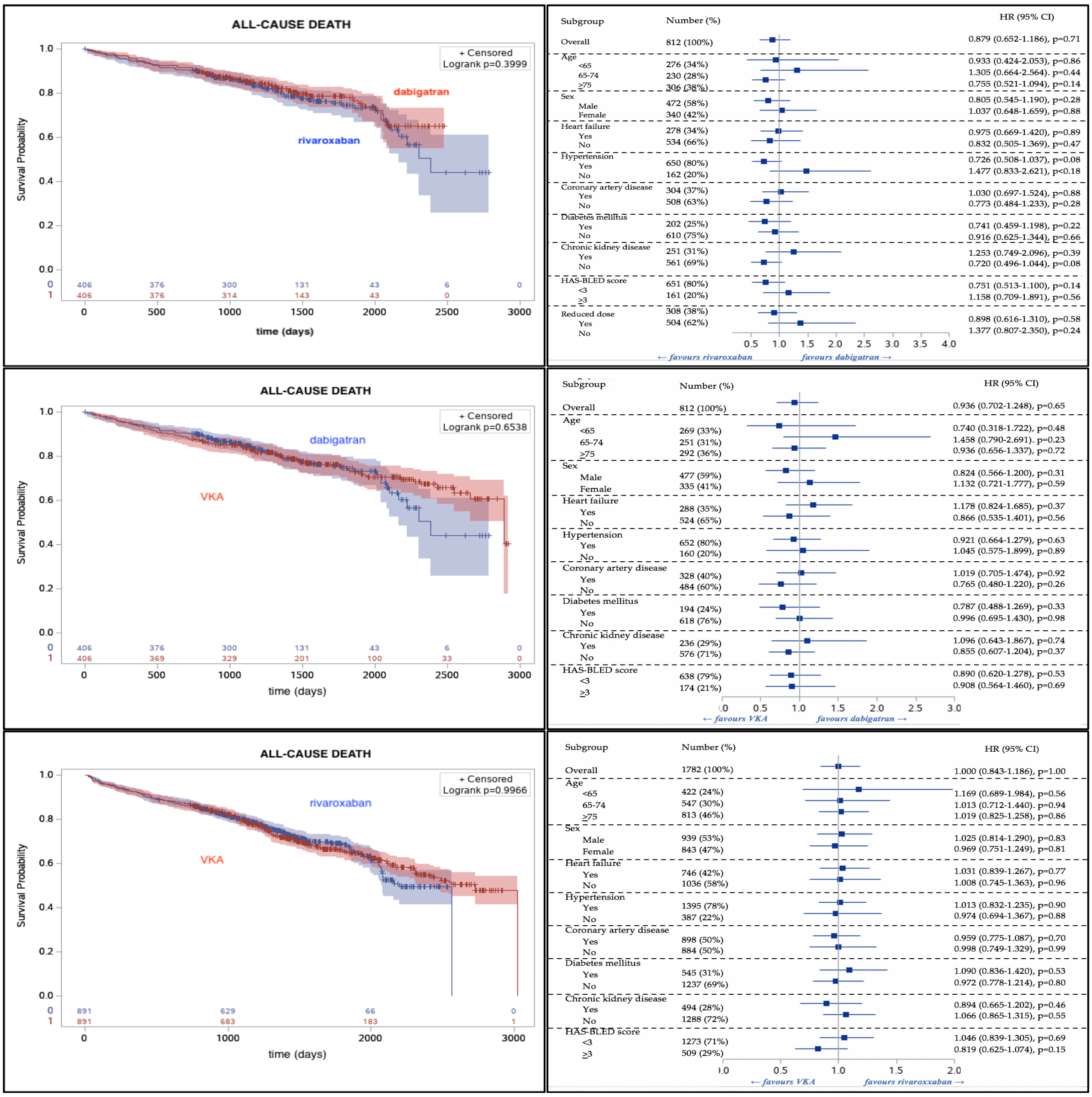

Abbreviations: VKA, vitamin K antagonist

b) thromboembolic events

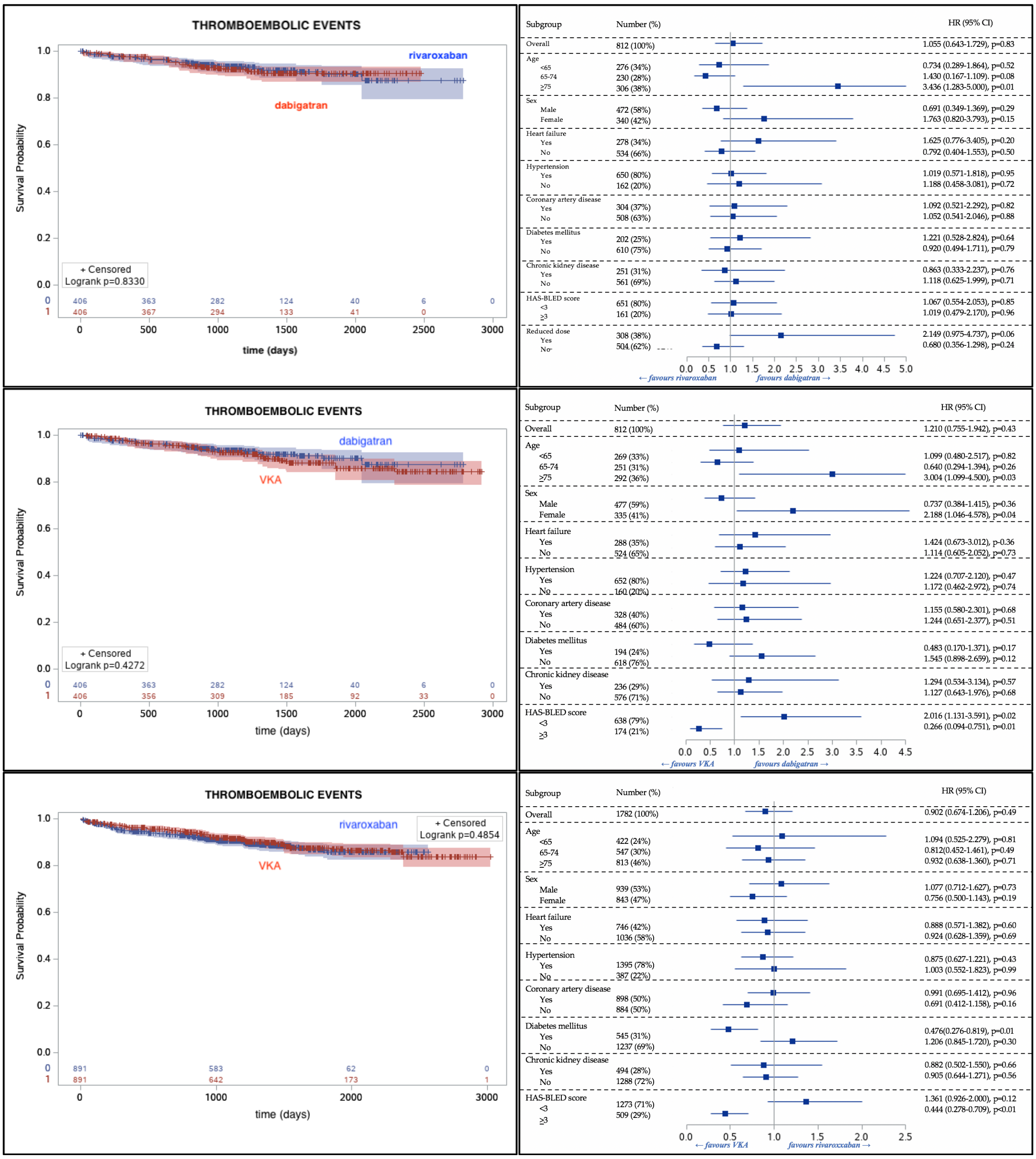

Abbreviations: VKA, vitamin K antagonist

c) hemorrhagic events

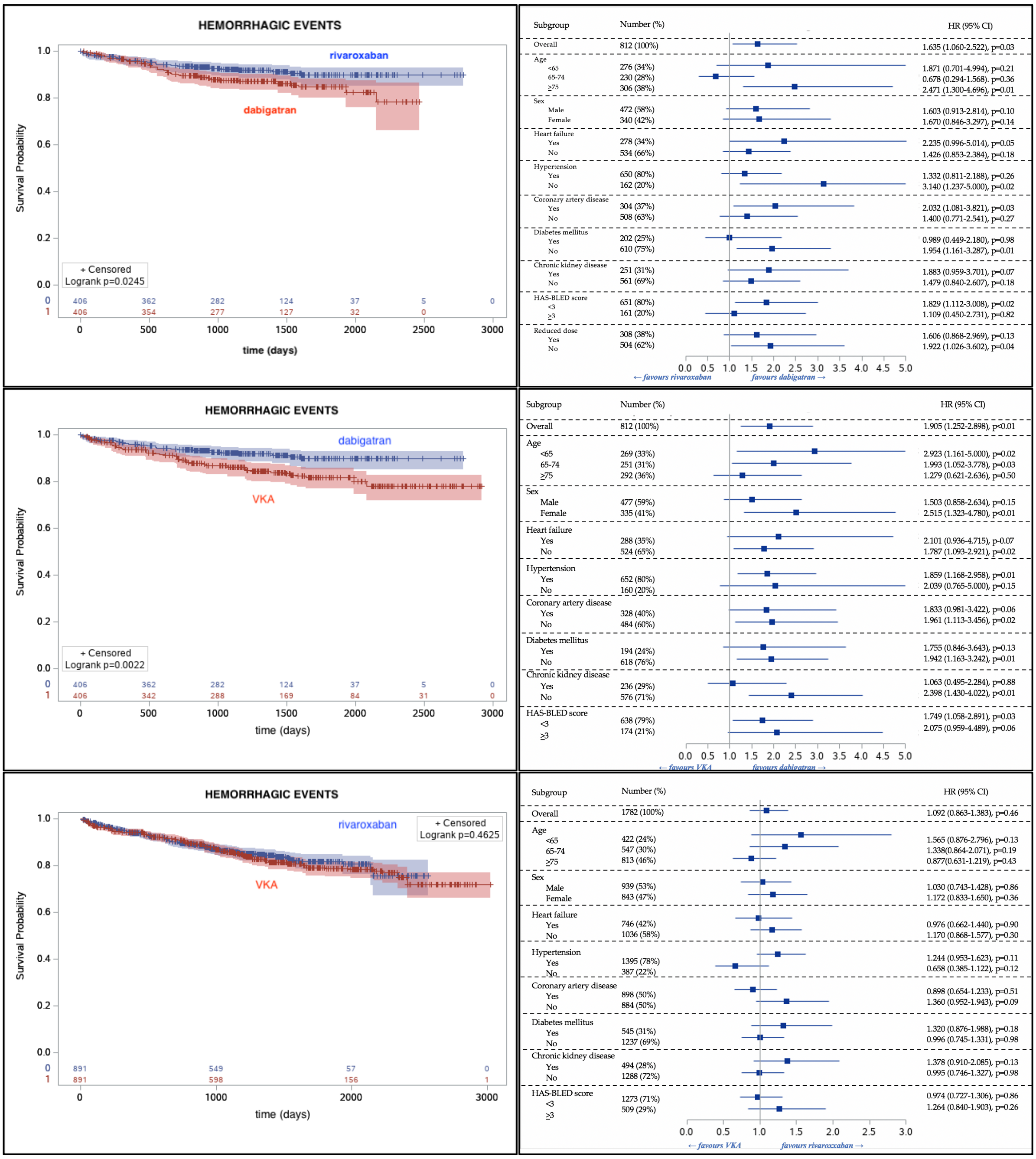

Abbreviations: VKA, vitamin K antagonist

Figure S2. Thromboembolic and bleeding risk scores and their predictive value.

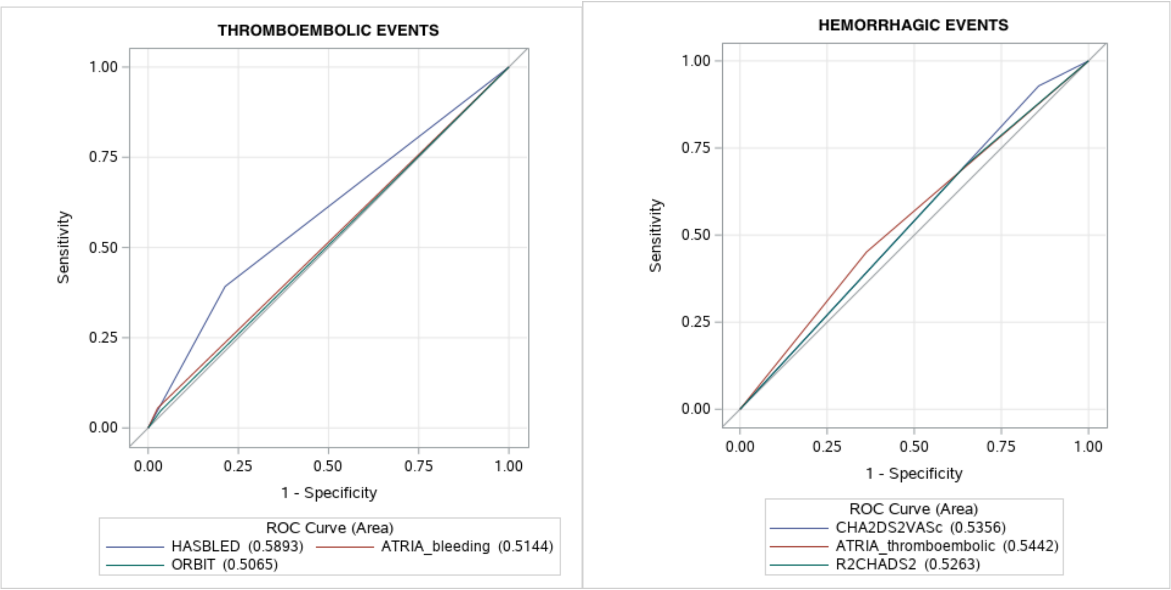

Table S1. Analyzed ICD-10 codes

**Ischemic events**	I63, I74.2, I74.3, I74.4, I74.5, I74.8, I74.9, I74, I65, I63.0, I63.1, I63.2, I63.3, I63.4, I63.5, I63.8, I63.9, I67.8, I67.9, I64, I74.0, I74.1 K55.0, M31.1, N28.0, H34.1, G45.8, G45.9, G45,
**Bleeding events**	I85.0, I84.4, I98.3, K22.1, K25.4, K26.4, K27.0, K29.0, K29.7, K31.8, K92.0, K57.3, K57.9, K62.5, K92.1, K92.2, K25.0, K25.2, K25.6, K26.0, K26.2, K26.6, K27.2, K27.4, K27.6, K28.0, K28.2, K28.4, K28.6, K29.2, K29.3, K29.4, K29.5, K29.6, K29.8, K55.2, K57.0, K57.1, K57.2, K57.4, K57.5, K57.8, K22.6, K66.1, K76.2, K62.6, K63.3, I62.0, I62.9, I60 , I60.0, I60.1, I60.2, I60.3, I60.4, I60.5, I60.6, I60.7, I60.8, I60.9, I61, I61.0, I61.1, I61.2, I61.3, I61.4, I61.5, I61.6, I61.8, I61.9, I62.1, I62, S06.4, S06.5, S06.6, D62, D68.3, D69.8, D69.9, H05.2, H11.3, H21.0, H31.3, H31.4, H35.6, H43.1, H44.8, H45.0, H47.0, H92.2, I23.0, I31.2, I71.3, J94.2, M25.0, M79.8, N02, N02.0, N02.1, N02.2, N02.3, N02.4, N02.5, N02.6, N02.7, N02.8, N02.9, N42.1, N42.1, N83.7, N85.7, N89.7, N92.0, N92.1, N92.3, N92.4, N92.5, N93, N93.0, N93.8, N93.9, N95.0, O71.7, R04, R04.0, R04.1, R04.2, R04.8, R04.89, R04.9, R23.3, R31, R31.0, R31.9, R58, R58 , S26.0, S27.1, T79.2, T81.0, Y60

Table S2. Comparison of rivaroxaban and dabigatran treatment groups after propensity score matching.

Variable	Rivaroxaban (n=406)	Dabigatran (n=406)	p value
**Demographics**			
Age (years)	70 [61-79]	69 [62-78]	0.35
Females	174 (43%)	166 (41%)	0.62
**Atrial fibrillation type**			
Paroxysmal	219 (54%)	208 (51%)	0.48
Non-paroxysmal	187 (46%)	198 (49%)	
**Comorbidities**			
Heart failure	129 (32%)	149 (37%)	0.16
Hypertension	324 (80%)	326 (80%)	0.93
Coronary artery disease	144 (35%)	160 (39%)	0.28
Diabetes mellitus	108 (27%)	94 (23%)	0.29
Ischemic stroke/TIA/ thromboembolism	56 (14%)	58 (14%)	0.92
Haemorrhagic events	31 (7.6%)	30 (7.4%)	1.00
COPD	21 (5.2%)	28 (6.9%)	0.38
Smoking	29 (7.2%)	32 (7.9%)	0.79
**Laboratory parameters**			
eGFR ≤14 ml/min/1.73m2	0 (0%) *n=346*	0 (0%) *n=353*	1.00
eGFR 15-29 ml/min/1.73m2	4 (1.2%) *n=346*	3 (0.8%) *n=353*	1.00
eGFR 30-49 ml/min/1.73m2	64 (18%) *n=346*	67 (19%) *n=353*	0.85
eGFR ≥50 ml/min/1.73m2	278 (80%) *n=346*	283 (80%) *n=353*	1.00

Number provided after in italic indicates the total number of patients available for that variable.

Abbreviations: COPD, chronic obstructive pulmonary disease; eGFR, estimated glomerular filtration rate; TIA, transient ischemic attack

Table S3. Comparison of vitamin K antagonist and dabigatran treatment groups after propensity score matching.

Variable	VKA (n=406)	Dabigatran (n=406)	p value
**Demographics**			
Age (years)	69 [62-78]	69 [62-78]	0.88
Females	169 (42%)	166 (41%)	0.89
**Atrial fibrillation type**			
Paroxysmal	221 (54%)	208 (51%)	0.40
Non-paroxysmal	185 (46%)	198 (49%)	0.40
**Comorbidities**			
Heart failure	139 (32%)	149 (37%)	0.51
Hypertension	326 (80%)	326 (80%)	1.00
Coronary artery disease	168 (41%)	160 (39%)	0.62
Diabetes mellitus	100 (25%)	94 (23%)	0.68
Ischemic stroke/TIA/ thromboembolism	45 (11%)	58 (14.3%)	0.21
Haemorrhagic events	21 (5.2%)	30 (7.4%)	0.25
COPD	31 (7.6%)	28 (6.9%)	0.79
Smoking	30 (7.4%)	32 (7.9%)	0.90
**Laboratory parameters**			
eGFR ≤14 ml/min/1.73m2	0 (0%) *n=358*	0 (0%) *n=353*	1.00
eGFR 15-29 ml/min/1.73m2	6 (1.7%) *n=358*	3 (0.7%) *n=353*	0.51
eGFR 30-49 ml/min/1.73m2	59 (16%) *n=358*	67 (19%) *n=353*	0.50
eGFR ≥50 ml/min/1.73m2	293 (82%) *n=358*	283 (80%) *n=353*	0.49

Number provided after in italic indicates the total number of patients available for that variable.

Abbreviations: COPD, chronic obstructive pulmonary disease; eGFR, estimated glomerular filtration rate; TIA, transient ischemic attack; VKA, vitamin K antagonist

Table S4. Comparison of rivaroxaban and vitamin K antagonist treatment groups after propensity score matching.

Variable	Rivaroxaban (n=891)	VKA (n=891)	p value
**Demographics**			
Age (years)	74 [65-81]	72 [65-80]	0.08
Females	420 (47%)	423 (47%)	0.92
**Atrial fibrillation type**			
Paroxysmal	467 (52%)	463 (52%)	0.89
Non-paroxysmal	424 (48%)	428 (48%)	0.89
**Comorbidities**			
Heart failure	381 (43%)	365 (41%)	0.47
Hypertension	689 (77%)	706 (79%)	0.36
Coronary artery disease	447 (50%)	451 (51%)	0.89
Diabetes mellitus	274 (31%)	271 (30%)	0.92
Ischemic stroke/TIA/ thromboembolism	157 (18%)	166 (19%)	0.63
Haemorrhagic events	98 (11%)	83 (9.3%)	0.27
COPD	114 (13%)	101 (11%)	0.38
Smoking	57 (6.4%)	55 (6.2%)	0.92
**Laboratory parameters**			
eGFR ≤14 ml/min/1.73m2	3 (0.4%) *n=831*	1 (0.1%) *n=829*	0.62
eGFR 15-29 ml/min/1.73m2	18 (2.2%) *n=831*	14 (1.7%) *n=829*	0.59
eGFR 30-49 ml/min/1.73m2	178 (21%) *n=831*	158 (19%) *n=829*	0.25
eGFR ≥50 ml/min/1.73m2	632 (76%) *n=831*	656 (79%) *n=829*	0.22

Number provided after in italic indicates the total number of patients available for that variable.

Abbreviations: COPD, chronic obstructive pulmonary disease; eGFR, estimated glomerular filtration rate; TIA, transient ischemic attack; VKA, vitamin K antagonist

Table S5. Distribution of direct oral anticoagulants according the recommendation for patients receiving reduced (A) and standard (B) dosing.

A. Reduced doses of direct oral anticoagulants		
**Variable**	**Dabigatran (n=177)**	**Rivaroxaban (n=379)**
**Correct prescription**		
**All**	**116 (66%)**	**250 (66%)**
**Recommended indications for dose reduction**		
- eGFR 30-49ml/min/1.73m2	101 (57%)	163 (43%)
- eGFR 15-49ml/min/1.73m2	24 (13%)	Not applicable
- Age ≥ 80 years	Not applicable	163 (43%)
- eGFR 30-49ml/min/1.73m2 + age ≥ 80 years	51 (29%)	Not applicable
**Indications to be considered (**≥ 2 below	26 (15%)	Not applicable
indications)	15 (8.5%)	87 (23%)
**Incorrect prescription**		
**All**	**52 (29%)**	**122 (32%)**
**Unknown reason**	28 (16%)	25 (6.6%)
**Indications to be considered**	24 (13%)	97 (26%)
- HAS-BLED ≥ 3	2 (1.1%)	5 (1.3%)
- Age ≥ 75 years	17 (9.6%)	83 (22%)
- NSAIDs and/or APT drugs	2 (1.1%)	7 (1.8%)
- Amiodarone	3 (1.7%)	2 (0.5%)
**Unknown appropriate prescriptions**		
**No data regarding CrCl or type of DHP-CCB**	**9 (5.1%)**	**7 (1.8%)**

B. Standard doses of direct oral anticoagulants		
**Variable**	**Dabigatran (n=224)**	**Rivaroxaban (n=502)**
**Incorrect prescription**		
**Recommended indications for dose reduction**	**26 (12%)**	**35 (7.0%)**
- eGFR 30-49ml/min/1.73m2	16 (7.1%)	Not applicable
- eGFR 15-49ml/min/1.73m2	Not applicable	35 (7.0%)
- Age $\geq$ 80 years	8 (3.6%)	Not applicable
- eGFR 30-49ml/min +age $\geq$ 80 years	2 (0.9%)	Not applicable
**Correct prescription**		
**All**	**153 (68%)**	**415 (83%)**
**Unknown appropriate prescriptions**		
**No data regarding eGFR or type of DHP-CCB**	**45 (20%)**	**52 (10%)**

Abbreviations: DHP-CCB – dihydropyridine calcium canal blockers, eGFR, estimated glomerular filtration rate; NSAID - nonsteroidal anti-inflammatory drugs

Table S6. Analysis of time-to-major adverse events, all-cause death, thromboembolic and hemorrhagic events for patients treated with appropriate and inappropriate reduced and/or standard NOAC doses.

	Major adverse event	All-cause death	Thromboembolic events	Haemorrhagic events
	HR [95% CI]	HR [95% CI]	HR [95% CI]	HR [95% CI]
Dabigatran (n=401)				
Incorrect dose	1.140 (0.733-1.772)	1.023 (0.600-1.744)	1.450 (0.619-3.398)	1.175 (0.510-2.708)
Correct dose	reference	reference	reference	reference
Dabigatran reduced (n=177)				
Incorrect dose	0.644 (0.382-1.087)	0.552 (0.302-1.012)	1.540 (0.434-5.459)	0.591 (0.196-1.782)
Correct dose	reference	reference	reference	reference
Dabigatran standard (n=224)				
Incorrect dose	1.715 (0.743-3.957)	1.674 (0.531-5.272)	1.742 (0.506-5.999)	2.393 (0.666-8.459)
Correct dose	reference	reference	reference	reference
Rivaroxaban (n=881)				
Incorrect dose	1.250 (0.969-1.613)	**1.624 (1.209-2.181)**	0.964 (0.554-1.675)	0.922 (0.572-1.486)
Correct dose	reference	reference	reference	reference
Rivaroxaban reduced (n=379)				
Incorrect dose	0.769 (0.567-1.043)	0.809 (0.573-1.143)	0.592 (0.301-1.164)	NA
Correct dose	reference	reference	reference	reference
Rivaroxaban standard (n=502)				
Incorrect dose	1.576 (0.927-2.681)	**2.754 (1.497-5.068)**	1.502 (0.537-4.195)	0.832 (0.280-2.658)
Correct dose	reference	reference	reference	reference
NOAC (n=1282)				
Incorrect dose	1.211	**1.429**	1.068	0.956

	(0.971-1.510)	**(1.104-1.848)**	(0.673-1.696)	(0.633-1.446)
Correct dose	reference	reference	reference	reference
NOAC reduced (n=556)				
Incorrect dose	**0.744 (0.572-0.967)**	**0.739 (0.549-996)**	0.740 (0.410-1.335)	0.644 (0.393-1.053)
Correct dose	reference	reference	reference	reference
NOAC standard (n=726)				
Incorrect dose	1.515 (0.971-2.364)	2.212 (1.293-3.783)	1.571 (0.716-3.447)	1.211 (0.971-1.510)
Correct dose	reference	reference	reference	reference

Abbreviations: CI, coincidence interval; NOAC, non-vitamin K oral anticoagulant; HR, hazard ratio
